# Anatomical Notes

**Published:** 1880-01

**Authors:** 


### ANATOMICAL NOTES.

NOTES ON THE NERVOUS SYSTEM OF A ZALOPHUS (CALIFORNIA SEA-LION).—A full-grown female Zalophus (see Pathological Notes) was found to present the following noteworthy features in its central nervous system: 1st, the great shortness of the spinal cord. This feature has been noticed in other Phocidae, and is still more pronounced in the Cetacea. In the present instance, the apex of the *Conus terminalis* was found opposite the articulation between the sixth and seventh dorsal vertebrae, and the length of the cord was about one-third of the length from the occiput to the end of the tail. There are noteworthy peculiarities in the arrangement and size of the cell groups in the anterior cornu, which will be carefully studied in a series of sections. The posterior white columns also present an anomalous arrangement. The Pons Varolii conceals the Trapezium from view. Oliviary bodies proper feebly marked. Cerebellum almost entirely covered; flattened, as in porpoise. Gyri numerous, Corpus Callosum only moderate (there is a still greater diminution in the porpoise and sperm-whale). Olfactory bulb well developed and hollow (it is atrophic in the earless seals and rudimentary in the porpoise). A noteworthy feature is the large size of the auditory nerve. This seems to be still larger in the porpoises. The hearing of seals is said to be very acute, and probably the development of this nerve is partly related to this and partly to the part it plays in maintaining the equilibrium of these marine animals.

---

THE ISLAND OF REIL IN THE PORPOISE.—The history of comparative cerebral morphology has been a repetition of erroneous dogmas, from its beginning to the present day. This is in great measure referable to the fact, that rarely has cerebral anatomy been studied by zoötomists so much for its own sake, as for the support of one side or other in a controversy.

Most of our readers may yet have a recollection of the Owen-Huxley dispute, in which one of the greatest English anatomists, Richard Owen, immortalized by the most careful and monumental researches on the extinct mammals of the Pampas, the giant birds of New Zealand, and Jurassic reptiles of England, was, in the zeal of discussion, betrayed into making the declaration that man alone had a posterior cornu and hippocampus. It was instantly shown by Huxley, that all monkeys, even including the Lemurs, possessed both. We have recently found a finely developed posterior cornu in the common porpoise.

Countless such errors have been committed; but there is one which has been perpetrated to the present day that we intend to refer more particularly to, inasmuch as on the erroneous assumption equally erroneous theories have been based.

It is believed, not among zoötomists, but by certain medical writers, that no animal exhibits as large and as well convoluted an Island of Reil as Man, and also that in this region and its neighborhood must be localized the function of speech for that reason.

The localization of the function of speech, or rather of the associated innervations on which it depends, may (from pathological experience most probably) have their main seat in the region indicated, but the reason, as quoted from the evidence afforded by comparative anatomy, is highly fallacious. The facts are quite opposed to such a deduction.

We have in a previous publication ("The Organology of the Island of Reil," *Journal of Mental and Nervous Diseases*, 1879) shown, that while the great atrophy of the Island in the seal, which we have since verified to be the same in the California sea-lion (*Zalophus Gillespiei*) lends countenance to the impeached theory, the development of this area in the hippopotamus is so great, that, according this theory, it should possess, if not speech, very complex symbolic faculties!

But the objection might be here made that the Island of the hippopotamus is not strictly homologous to that of man. Simply stating for the present, that the exuberantly developed island of the hippopotamus, which is relatively three times as large as that of the horse, being homologous to the latter, must be, therefore, homologous to that of man (since it is admitted that the equine and human islands correspond), we will now refer to another, still more damaging fact to the deduction in question.

We dissected the brain of a porpoise, obtained from the New York Aquarium this July, and were astonished to find an Island of Reil twice as extensive and with four times as many convolutions as the human average brain shows. This region is triangular, with its longest side convex towards, and following the convexity of the hemisphere. The two shorter sides were so directed that the angle formed by their meeting was downwards. The longest of the short sides was in front, and met the superior contour at a very sharp angle. The shortest posterior side ran upwards and backwards. The Island of Reil is *absolutely covered by the overlapping operculum and temporal lobe*, more decidedly so than in the elephant, and in this respect corresponding to man and the anthropoid apes

The gyri having the same direction as in man, are dovetailed as in the latter, with corresponding gyri on the inner face of the operculum. They are so regularly arranged as to simulate the corrugations of corrugated iron, differing from the human in exhibiting no subdivisions. There is also this difference—the human Island is highest in front and gradually tapers down as we go posteriorly; in the porpoise it is just the opposite.

If we place side by side the following brains, we shall perceive an almost uninterrupted gradation: Bear (better still coatimondi), otter, sea-lion, earless seals (*Callocephalus groenlandica*), and porpoise. The transformation may be signalized as follows: 1st. The hemispheres become broader on account of the increasing disproportion of the cranial diameters. 2d. The cerebellum becomes flatter and more completely covered. 3d. The Pons Varolii conceals the Trapezium. 4th. The olfactory lobe atrophies. 5th. The Corpus Striatum diminishes.

This progression is the more noteworthy, as in the light of the Evolution doctrine (we say "doctrine," not "theory," for we can readily afford to disregard the senile lucubrations of even a Virchow in opposition), the Cetacea, or whale-tribe have most likely developed from seal-like creatures, while, on the other hand, the transition from the earless to the eared seals, and from these to the otter, is very easy. The whale is in fact the terminus of an aberrant twig from the phyllogenetic ancestral tree of the Carnivora.

Now, while there is this gradual metamorphosis of the brain, we find that the Island of Reil cannot be included in it. In fact, although it rises in this respect that it becomes more and more completely covered as we pass up in the series, as regards area and the development of gray matter it sinks from the bear to the seal, to rise again in the porpoises (and whales?) to the highest degree of development known to obtain in the entire animal series.

In explaining, on the basis of mere speculation, the atrophy of the seal's Island, we suggested the correlated atrophy of the hypoglossal and facial peripheries, the facial muscles being atrophic, and the mobility of the tongue limited. But on this purely theoretical ground, the porpoise, with these peripheries still less developed, ought to have a still more atrophic Island, whereas it has the highest development attained. There is one group of innervations, which is developed in the porpoise (all *cetacea*), to a degree not even remotely approached by any other animal. These are innervations, which, from their physiological association with those of the tongue and face, we should predicate an identical or closely neighboring cortical field for, assuming the doctrine of cortical localizations to be correct. We refer to the muscles of the larynx and blow-hole.

The blow-hole muscles belong to the territory governed by the facial, those of the larynx to the spinal accessory. Their innervations must be of a complex character, when it is recollected that the larynx of the purpoise is prolonged as a free tube, passing vertically up through the pharynx to reach the blow-hole.

But all these suggestions are purely tentative and theoretical. We believe, however, that so far as the localization doctrine is admissible, that a careful and judicious comparison of animals possessing remarkable peripheries will do much to support and extend it.

E. C. SPITZKA, M. D.

